# Flow and temporal effects on the sonolytic defluorination of perfluorooctane sulfonic acid

**DOI:** 10.1016/j.ultsonch.2023.106667

**Published:** 2023-10-30

**Authors:** Tim Sidnell, Angel J. Caceres Cobos, Jake Hurst, Judy Lee, Madeleine J. Bussemaker

**Affiliations:** a School of Chemistry and Chemical Engineering, University of Surrey, Guildford, Surrey GU2 7XH, United Kingdom; b ARCADIS, 1 Whitehall Riverside, Leeds LS1 4BN, United Kingdom

**Keywords:** PFAS, Sonolysis, Solvated electron, Ultrasonic degradation, Recirculating, Flow

## Abstract

The removal of per- and polyfluoroalkyl substance (PFAS) pollution from the environment is a globally pressing issue, due to some PFAS’ recalcitrant, bioaccumulative, and carcinogenic nature. Destruction via ultrasonic waves (sonolysis) is a promising contender for industrialisation due to; moderate power consumption, applicability to several PFAS and sample types, and limited by-products. Liquid flow rate through an ultrasonic reactor can affect the size, shape, and spatial distribution of ultrasonic cavities and hence their chemical activity. Such effects have not been studied during PFAS sonolysis, and temporal effects have not been studied much beyond the reactant concentration. Here, the effects of varying recirculating flow rate on the ultrasonic defluorination of perfluorooctane sulfonic acid (PFOS) and implications for industrial scale up are presented. Under the ultrasonic power (200 W L^−1^, 2.27 W cm^−2^) and frequency (410 kHz) used, flow rates of 79 and 214 ml min^−1^ enhanced defluorination up to 14 % during 30 min of treatment. However, these effects were temporal and most significant in the initial minutes of treatment. This indicated a dynamic bubble size distribution which stabilised after around 15 min. Defluorination rates of PFOS were compared with measured potassium iodide dosimetry, calorimetry, sonoluminescence (SL), and sonochemiluminescence (SCL). Flow rates which enhanced defluorination correlated moderately with enhanced SCL and negatively impacted SL, calorimetry, and dosimetry. Effects were attributed to perturbed cavity surfaces, leading to asymmetric cavity collapse, and the possibility of enhanced solvated electron production/interaction. SL, SCL, dosimetry, and calorimetric measurements were also temporal, and each showed different times to equilibrate. Flow rates of 439 and 889 ml min^−1^ returned all sonochemical measurements to the levels without flow, likely due to continued collapse temperature quenching by furthered bubble asymmetry. Flow also enhanced reactor cooling, which is significant for industrial temperature control. The pump energy consumed was small (≈1.9 %) compared to that of the amplifier and chiller, hence, PFOS defluorination was more cost-effective using flow. However, the effect may be limited for the longer treatment times needed for environmental remediation.

## Highlights

• Investigation of flow effects for PFAS treatment presented.
• Temporal PFOS defluorination and sono-chemical/thermal effects observed.
• Flow likely increased asymmetry of bubble collapse.
• Solvated electron release was likely modified with asymmetric bubble collapse.
• Flow ultimately reduced the cost of PFOS treatment.

## 1 Introduction

The remediation of per- and polyfluoroalkyl substances (PFAS) by ultrasound has been studied in at least 32 works to date [1] and critically reviewed elsewhere [1], [2], [3], [4], [5], [6]. Sonication mineralises PFAS into comparatively harmless inorganics, including; F^−^, SO_4_^2−^, CO_2_, and H^+^ with limited amounts of short chain PFAS formed and subsequently degraded [1], [7], [8], [9]. PFAS mineralisation via sonolysis has been demonstrated in many aqueous media, ranging from pure PFAS dissolved in water [7], [9], [10], [11] to PFAS in landfill leachate [12], [13], and aqueous film forming foams (AFFFs) [14], [15], [16]. Thus, several sonolytic rate controlling parameters have been assessed including; PFAS structure [17], concentration [10], ultrasonic power, [18] ultrasonic frequency [9], [19], [20], and pH [18], [21]. While continuous recirculation of aqueous PFAS has been used in research [11], [14], [22], [23], the speed of flow has not been considered for its effects on reaction rates. Flow can be applied using; impellors, recirculating pumps, or (in the case of acoustic- and micro-streaming) by the ultrasonic transducer itself [24] and is known to influence, and be influenced by, ultrasonic cavitation bubbles [24], [25], [26], [27], [28]. The flow of liquid around an ultrasonic bubble can affect sonochemical activity, measured via sonochemiluminescence (SCL) or potassium iodide (KI) dosimetry [27], [28], [29], [30], [31], [32], [33], which in turn can affect chemical degradation rates [26], [32], [33], [34], [35]. Despite several experimental and modelling works to date considering flow effects, like many sonochemical parameters, the effects of flow are not simple nor well understood [27]. Flow effects are specific to the reactor and chemical set-ups being considered and can show augmentation, diminishment, a combination of both, or no effect on reaction metrics, as observed experimentally or theorised (Table 1). These effects depend on the mode of flow application, flow rate, frequency, power, surface stabilisation, fluid volume, and other factors [28], [29], [32], [35], [36]. Hence, an experimental work is a reliable and easy approach to understand flow effects on PFAS treatment.

**Table 1:** Factors theorised and observed to be affected by increasing mechanical flow rate in sonochemical reactors.

Augmented by flow	Diminished by flow	Show an optimum flow
Bubble antinode attraction [36]*	Secondary Bjerknes forces [33]^+^, [36]*	SL and SCL [36]*
Sonochemically active region [27], [34]*	Bubble agglomeration [33]^+^, [36]*	Radical production [24], [28], [29]*
Bulk mixing [35]*	Collapse symmetry [37]*	
Collapse asymmetry [37]*	Bubble degassing [38]^+^	
Jet injection [37]*	Collapse temperature [37]^+^	
Non-volatile species injection [37]*		
Generation of new nuclei [35]^+^		
Microstreaming [35]^+^		
	Key:	[Observed]*
		[Theorised]^+^

Flow effects have largely been studied in ultrasonic reactors in which the transducer only partially covers the reactor diameter [25], [27], [33], [35], [38], [39]. Partial cross-sectional coverage by the transducer initiates high positive flow rates in the direction of sound propagation (above/below the transducer/horn respectively), while negative flows occur outside this region. This specific type of bulk convective flow [25], [27], [39] does not occur with total width coverage [28], [29] and thus obscures the effects of external flow addition. Flow has also been studied under indirect sonication (transducer outside of reactor) [34], which is not compatible with the typical direct sonication used for PFAS sonolysis (and other contaminants) [1], [7], [9], [19], [40], [41], [42], [43]. Further, while an impeller can enhance sonochemical activity, there is an associated activity loss in the region where the impellor sits [27], [29] and prior work has concluded that recirculation is preferred over mechanical stirring to enhance sonochemical activity [28]. Hence, the findings of recirculating flow effects on PFOS sonolysis are presented for transducers which directly sonicate the entire reactor diameter. While tests at different powers and frequencies might be of scientific interest, optimisation of frequency [9] and power (under review) suggests that this would not aid the development of a high performance PFAS sonolysis reactor.

## 2 Materials and methods

The materials and methods used in this work were similar to those in previous research [9], [30], [31], [32]. ≥3 repeats were completed for all experiments, so that average values and standard deviations are presented in the results.

### 2.1 Chemicals

≥99 % Ammonium molybdate tetrahydrate, 98 % heptadecafluorooctanesulfonic acid potassium salt (PFOS), ≥99.9 % HPLC grade methanol, 99.5 % reagent grade potassium iodide (KI), analytical standard 0.1 M sodium fluoride, and 97 % luminol (5-amino-2,3-dihydro-1,4-phthalazinedione) were purchased from Sigma-Aldrich®. Ionic strength adjustor (ISA) TISAB I was purchased from Cole-Parmer. 98.3 % sodium hydroxide was purchased from Fisher Chemical. All solutions were made using distilled water (Milli-Q) from an Elix Essential 3 (UV) Type 2 device operating at 18.2 MΩ cm.

### 2.2 Reactor and electronic configuration

The aqueous PFOS solution was sonicated in a glass jacketed 0.6 L reactor as previously described [30]. The liquid sat within a tubular cavity in the reactor with a diameter of 6.7 cm. The reactor fluid was either without flow (400 ml) or circulated using a closed tube system and a peristaltic pump operating at 10–100 % of the available output speed (total 420 ml). The circulation volume of 420 ml was used to maintain the liquid height for experiments with and without flow. The pump output was calibrated using a volumetric cylinder and stopwatch, as shown in Fig. S1 (Supplementary information). The pump was operated at 79-, 214-, 439-, and 889 ml min^−1^ (10, 25, 50, and 100 % of the pump speed, respectively). The reactor was filled with 400 ml of 10 mg L^−1^ PFOS solution, as per our previous work [9], plus a further 20 ml to account for the fluid in the pump tubing. 10.0 °C Cooling water (CW) was supplied to the reactor jackets using a 20 L Cole-Palmer PolyScience MX open bath circulating chiller. Note that the chiller did not have an external thermocouple, so only the CW temperature was directly controlled. Further, the presence of a thermocouple in the reactor would have disrupted the ultrasonic wave motion and fluid flow. CW maintained the reactant solution between 18.3 and 26.4 °C (Table S1). The CW was pre-cooled prior to experiments and measurement of power consumption. A 400 kHz transducer was used in all experiments, as previously optimised for PFOS degradation [9], however, it was operated at 410 kHz since this provided minimum reflected power (indicated on the amplifier). The transducer was purchased from Honda electronics Co. LTD. and comprised a 5 cm diameter PZT piezo-electric ceramic connected to a 10.0 cm diameter stainless steel plate. The transducer was attached to the base of the reactor, creating a direct contact area of 35.26 cm^2^ with the liquid. Power was supplied to the transducer using an AG 1006 T&C Power Conversion amplifier. The impedances of the amplifier and transducer were matched using a low frequency RF impedance step-up transformer (SUT) operated as a step-down transformer and static was discharged through a 10 kΩ resistor, used to ground the line.

### 2.3 Sonication, sampling, and cleaning procedures

In all PFOS experiments, 10.0 ± 1.0 mg L^−1^ aqueous PFOS was sonicated for a total sonication time of 30 min. Note that this concentration exceeds bubble saturation conditions such that minor changes in initial concentration will not affect the zero order degradation rate [1], [7], [10]. During sonication, the ultrasound was switched off for 1 min, every 5 min, to measure the solution temperature and gather a 2.00 ml sample for fluoride analysis (Table S1). A total of 10 ml (∼2 %) of the solution was removed during sampling, this difference was insignificant compared to the difference in fluoride concentrations between timepoints (see Supporting Data File). The sample was collected using a 5.00 ml micropipette and disposable 5.00 ml polypropylene pipette tips. Following experimentation, all glassware used were washed three times with methanol to remove any residue and desorb PFOS which may have adsorbed to the glass [44]. Between measurements, the thermocouple and fluoride electrode (Section 2.4.4) were both washed with Milli-Q water and blotted dry using lint-free tissue.

### 2.4 Reaction characterisation

#### 2.4.1 Power characterisation and consumption

The load power supplied to the transducer was set via the amplifier and balanced against reflected power such that Equation (1) holds:

(1) $$\mathrm{Forward}\;power\;\left(FP\right)=\;Load\;power\;\left(LP\right)+Reflected\;power\;\left(RP\right)$$

The calorimetric power ($P_{\mathrm{Cal}})$ was calculated using a previously derived method [45] which was modified to account for heat losses through the reactor walls (Eq. (2)). Heat loss was accounted for to appreciate any calorimetric differences and impacts that flow rate may have on heat transfer in different flow regimes, to inform the overall calorimetric coupling efficiency. The calorimetric power was calculated for readings taken every 5 min and averaged over the 30 min treatment time.

(2) $$P_{\mathrm{Cal}}=mC_{p}\frac{\mathrm{dT}}{\mathrm{dt}}+\;{\left(P_{\mathrm{Cool}}\right|}_{\overset{¯}{T}}$$

Where:

$P_{\mathrm{Cal}}=$ Calorimetric power (Watts).
m= Mass of water (g).
$C_{p}=$ Specific heat capacity of water (taken as constant, 4.18 J g^−1^ K^−1^) [46].
$\frac{\mathrm{dT}}{\mathrm{dt}}=$ Temperature (T) change during time $t_{0}$ to $t_{1}$, = $\frac{T_{1}-T_{0}}{t_{1}-t_{0}}$ (K s^−1^).
${\left(P_{\mathrm{Cool}}\right|}_{\overset{¯}{T}}\left(=mC_{p}\frac{dT_{\mathrm{Cool}}}{dt_{\mathrm{Cool}}}\right)=$ Cooling power (W) i.e., the rate of heat loss through walls of reactor at evaluated at the average temperature, $\overset{¯}{T}=\frac{T_{1}-T_{0}}{2}$.

$P_{\mathrm{Cool}}$ was determined as a function of temperature and pump output by monitoring the temperature change over time for 420 ml of water, with an initial temperature of 80.0 °C, added to the reactor, without sonication. The temperature was measured every 5 min and cooling powers relative to temperature change calculated for flow rates of 0, 79-, 214-, 439-, and 889 ml min^−1^ (0, 10, 25, 50, and 100 % of the pump speed, respectively). The temperatures measured during calorimetry and the associated cooling functions are plotted in Fig. S2. The calorimetric coupling efficiency, $\eta_{\mathrm{Cal}}$, between applied (load) power and calorimetric activity, was calculated using Eq. (3).

(3) $$\eta_{\mathrm{Cal}}=\frac{P_{\mathrm{Cal}}}{\mathrm{LP}}$$

The amplifier and chiller electrical power usage was measured with a Prodigit Electronics Co. Ltd. Model 2000MU Plug-in power monitor (UK version) to ±0.01 kWh [47]. The power consumed by the amplifier, chiller, and pump during 30 min of sonication were 0.32-, 0.21-, and, 0.01 kWh, respectively.

#### 2.4.2 KI dosimetry

Hart and Henglein’s dosimetry method [48] was used to indicate the relative concentration of radical species formed during sonication. 0.1 M KI and 0.5 mM ammonium molybdate tetrahydrate solution was sonicated and 2.00 ml samples taken every ten minutes for 30 min. The absorbance of 350 nm light by the samples was measured using an Evolution 201 UV–vis spectrometer and a 1.0 cm wide quartz cuvette. The absorbance was saturated after only a few minutes of sonication due to I_3_^−^ production. Hence, the samples were diluted 5.0 x prior to measurement to obtain differentiable results. The I_3_^−^ concentration was then calculated from Eq. (4), according to the Beer-Lambert law [45], [49], modified to account for the dilution applied.

$$C=\;D\frac{A}{\varepsilon l}$$

Where:

A= Absorbance of the sample (Arbitrary units).
ε= Molar absorptivity coefficient of I_3_^−^ ions (26,303 L mol^−1^ cm^−1^) [45].
C= Concentration of I_3_^−^ ions (Mols L^−1^)
D=dilutionfactor(5.0)
l= Length of light path (cuvette width = 1.0 cm)

Note that, in air saturated systems, this method can lead to inaccurate absolute radical concentration measurements, due to the formation of nitric acid from N_2_ which can oxidise KI [50] and catalysation of the reaction by oxygen [51], [52]. Using ammonium molybdate tetrahydrate enhances the sensitivity to hydrogen peroxide, reducing this interference. Regardless, the application of dosimetry in this case is used to investigate the relative radical production between different settings and values presented here may not be comparable to those from others.

#### 2.4.3 Sonochemiluminescence/sonoluminescence image analysis

**Fig. 1:**
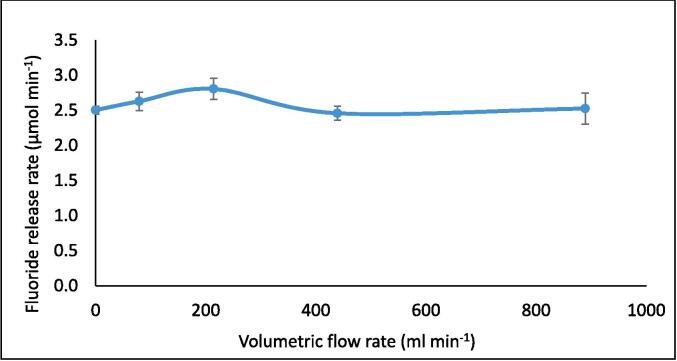
Variation of fluoride release rate (μmol L^−1^ min^−1^) with increasing recirculating flow rate (ml min^−1^) during PFOS sonolysis under 410 kHz and 80 W ultrasound, with cooling water applied. The solution was sonicated for 30 min with fluoride samples taken every 5 min, with a zero-order rate achieved. Error bars represent the standard deviation of three repeats.

Sonoluminescence (SL) and sonochemiluminescence (SCL) light emissions were captured using an ANDOR iXon3 EMCCD camera and associated ANDOR software. The camera was placed adjacent to the reactor, inside a light-sealed box within a dark room. SL emissions were assessed by sonicating 420 ml of Milli-Q water at room temperature (19.0 °C, without CW supplied) while the camera was operated at −70 °C with an electron multiplying (EM) gain of 50 and exposure time of 15.0 s. SCL emissions were similarly assessed by sonicating a 420 ml solution of 0.1 M NaOH and 1.0 mM luminol using an EM gain of 4 and exposure time of 2.0 s. The SCL EM gain and exposure time were reduced compared to SL due to the greater level of SCL light emission, which would otherwise saturate the images. Additional SL/SCL images and measurements were made using a tubular glass reactor with the same internal diameter as the reactors shown in Fig. 1 but without an attached cooling jacket or inlet/outlet ports, for consideration of the no-flow case. The ultrasound was turned on 30 s prior to image capture.

#### 2.4.4 Fluoride analysis

The release of fluoride ions correlated well with PFOS degradation in previous work and the same device (a Cole-Parmer Combination Fluoride Ion Selective Electrode) and methodology were used here [9], [53]. The probe contains a filter which only allows F^−^ ions to be assessed but is moderately affected by pH changes [53]. The probe was calibrated by insertion, at constant depth, into a series of aqueous NaF solutions (10^−6^–10^−1^ M). The solutions were magnetically stirred at 100 rpm and temperature controlled using a heated plate at 25.0 ± 0.05 °C. A volume of ionic strength adjustor equal to 10 % of the fluoride sample was then added, and conductivity was recorded after the reading stabilised (indicated on the conductivity meter). The correlation between [F^−^] and solution conductivity is shown in Fig. S3. Note that the pre-exponential coefficient derived is lower than in previous work [9] due to aging of the electrode [53]. The same procedure was applied when analysing samples from the reactor and the conductivity readings converted to [F^−^] using the derived correlation.

## 3 Results and discussion

### 3.1 Effects of flow on PFOS defluorination

Under the recirculation rates studied (0–889 ml min^−1^), flow had a moderate (up to +14 %) impact on the PFOS ultrasonic defluorination rate during 30 min of sonication (Fig. 1). The peak at 214 ml min^−1^ was statistically significant (P < 0.05, as determined by the *t*-test) compared to fluoride release rate at 0- and 439 ml min^−1^. The statistical significance to the peak was not as noticeable (P < 0.15) for flow rates 79- and 889 ml min^−1^. Hence, an optimised flow rate could provide 14 % faster treatment times compared to non-optimised flow or batch systems. This would reduce operating times and costs at full scale and enable the development of a flow through- or recycle-system for the treatment of PFAS contaminated waters. The presence of a maximum rate suggests competition between physiochemical phenomena as the flow rate is increased and is consistent with similar prior research [28], [33]. Further, it suggests the trend is not simply an effect of the relative residence times (per pass) experienced by the process fluid, as confirmed in Fig. S4. This is expected for a recirculating flow in a closed loop since, for a fixed reactor volume and different flow rates, the total time the fluid spends in the recirculating reactor, for a given treatment time is independent of flow rate. Rather, the total residence time depends on the ratio of reactor volume (here 400 ml) and reactor volume plus recirculation system volume (here 420 ml). Hence, for all flow rates used here, this ratio is 0.952:1.00, and for 30 min sonication each test achieved a residence time of 28 min and 34.3 s. To confirm whether the peak is phenomenologically significant and understand why it occurs, the impact of flow rate on calorimetry, SL, SCL, and KI dosimetry is assessed in the next section and compared with the F^−^ release rates.

### 3.2 Comparison with sonochemical metrics

**Fig. 2:**
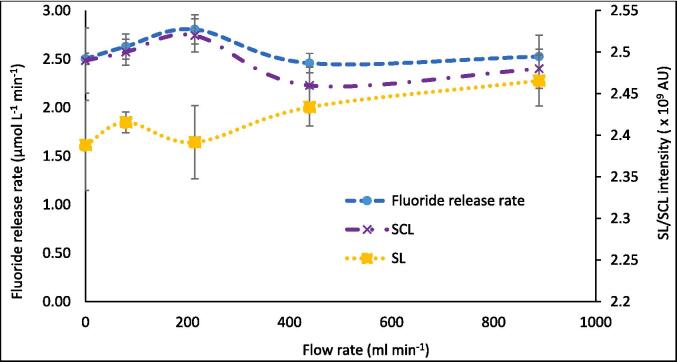
Variation of F- release rate (μmol L^−1^ min^−1^), SL intensity (AU), and SCL intensity (AU) with recirculating flow rate (ml min^−1^). Note the none zero Y-axis baseline for S(C)L data. Data taken under 410 kHz and 80 W ultrasound. Error bars represent the standard deviation of three repeats.

**Fig. 3:**
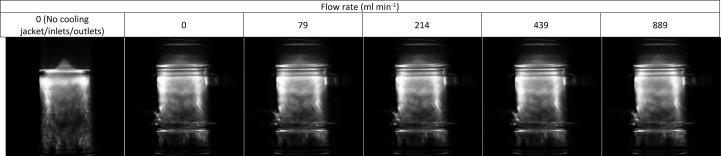
Representative sonoluminescence images for sonolysis of 420 ml of Milli-Q water using 410 kHz ultrasound at 80 W applied power, at liquid flow rates of 0, 0, 79, 214, 439, and 889 ml min^−1^. The image most left shows a reactor without flow, inlets, outlets, or cooling jacket, which enables the standing wave under no flow to be seen more clearly.

**Fig. 4:**
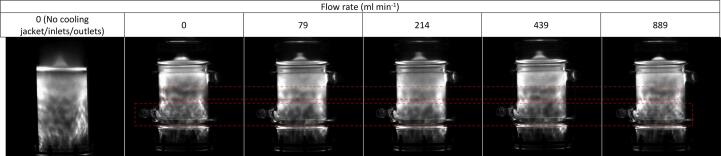
Representative sonochemiluminescence images for sonolysis of 420 ml of Milli-Q water using 410 kHz ultrasound at 80 W applied power, at liquid flow rates of 0, 0, 79, 214, 439, and 889 ml min^−1^). The image most left shows a reactor without flow, inlets, outlets, or cooling jacket, which enables the standing wave under no flow to be seen more clearly. The upper red dashed box shows the blending of a dark band with light regions into a grey band at intermediate flow rates. Similarly, the lower red dashed box shows several light and dark regions agglomerating into a few larger light and dark regions at intermediate flow rates.

**Fig. 5:**
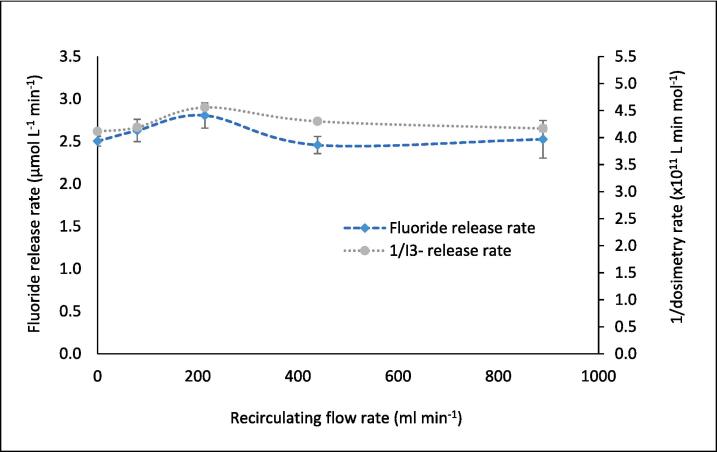
F- release rate (μmol L^−1^ min^−1^) and inverse of radical production rate (L min mol^−1^) vs recirculating flow rate (ml min^−1^). Data taken every 5–10 min for 30 min sonication under 410 kHz and 80 W applied power with cooling water applied. Error bars represent the standard deviation of three repeats.

Both SL and SCL intensity values changed little (3.25 % and 3.39 %, respectively) with increasing volumetric flow rate, showing significant error bar overlap in (Fig. 2). Similarly, the SL/SCL images captured at each flow rate (Fig. 3 and Fig. 4) show little variation between conditions used. By increasing the SL/SCL data baseline (Fig. 2), the relative trend of SL and SCL intensity is exacerbated. This demonstrates average SCL data with a similar relative trend and statistical overlap to those of F^−^ release with increasing flow rate (Fig. 1) and SL data with an almost inverse trend. However, the SCL intensity error at 214 ml min^−1^ does overlap with that at 0 ml min^−1^, unlike for fluoride release rate. Thus, there is a dependence of SCL on flow rate, which correlates with proportional, yet stronger, effects on PFOS defluorination rate. KI dosimetry was used to measure the relative production of oxidsing radicals. Like SL intensity, the inverse of radical production rate fit well to the trend of fluoride release rates (Fig. 5). So, KI dosimetric measurements were inversely proportional to SCL under flow, but proportional to SL. This agrees with other work that radicals are not responsible for PFOS sonolysis [1], [7], [9].

**Fig. 6:**
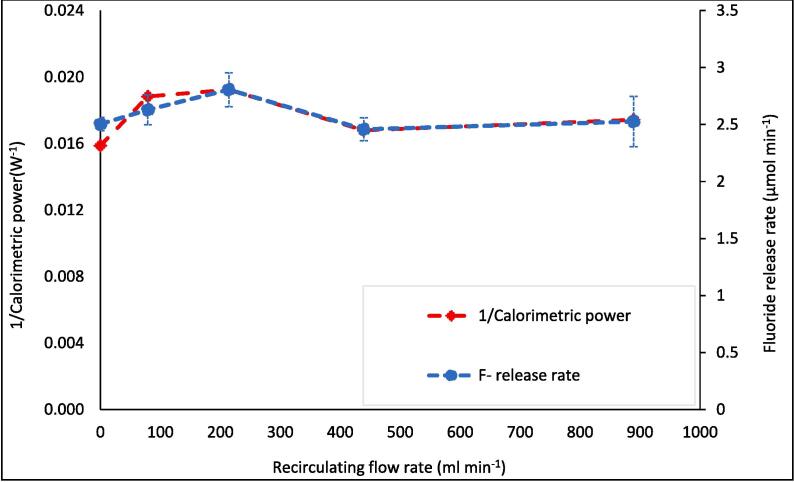
Variation of 1/[calorimetric power] (W) and F- release rate (μmol L^−1^ min^−1^) with increasing recirculating flow rate (ml min^−1^). Data taken every 5 min for 30 min sonication under 410 kHz and 80 W applied power. Error bars show the standard deviation of three repeats.

The SL/SCL images show moderate changes in areas of activity and inactivity with increasing flow (Fig. 3, Fig. 4). Most significantly, the upper half of the reactor in the SCL images shows (at no flow) a dark band across the width of the reactor and some lighter regions above and to the left (Fig. 4, labelled with the upper red dashed box). These regions intersperse under moderate flow (79–214 ml min^−1^), and the top of the reactor becomes homogenised and grey in colour. This trend is reversed as flow rate increases beyond 214 ml min^−1^. Similarly, under no flow, the region parallel to the liquid inlet (highlighted with lower red dashed box) is heterogeneous with small regions of black and white “mottled” throughout. These regions of activity and inactivity are likely due to quasi-acoustic streaming (bulk liquid flow) caused by the transducer [24]. When increasing flow from 0 to 214 ml min^−1^, these regions agglomerate into larger, less populous, regions of black and white. This trend is also reversed as the flow increases beyond 214 ml min^−1^. Deformation of these regions at intermediate flow rates and (re)formation at high and low flows qualitatively agrees with the trends in fluoride release rate, SL, SCL, dosimetry, and calorimetry (Fig. 6). This suggests that there are, indeed, underlying phenomena which explain the maxima and minima in fluoride release rate and other metrics. The correlation of SCL but not SL with PFOS defluorination agrees with previous work, which compared these values under varied ultrasound frequency [9]. This gives further evidence that PFAS are predominantly degraded via a sonochemical (as opposed to a sonothermal) mechanism [1], [9]. Further, the limited flow effects on SL/SCL, with a minimum/maximum at around 220 ml min^−1^, agree with prior work using the same experimental set up [32]. This suggests reliability of the results and that the underlying phenomena are similar under differing conditions (300 kHz, 40 W [32]), with some predictability of the optimum/suboptimum flow rates for SL/SCL.

The blurring of light and dark SCL regions has been noted previously with the addition of flow via overhead stirring [27]. The presence of streaming lines in the reactor base, bright sonochemical activity at the liquid surface, and increased contrast of sonochemical images under similar mixing rates (>214 ml min^−1^) also confirm findings at 376 kHz, using an overhead stirrer [29]. In a similar experiment, recirculating flow was proposed to enhance the traveling wave component in a sonochemical reactor [28]. This may partly explain the homogenisation of white and black regions in Fig. 4, as sonochemically active and inactive bubble were interspersed throughout the liquid under a travelling wave, rather than active bubbles being locked in position at the antinodes. Based on this, one would expect homogenisation to continue with higher flow rates, and hence more effective bulk mixing, which does not occur. Further, even with no flow, the standing wave component (shown most clearly in the left most images in Fig. 3 and Fig. 4) appears relatively weak. This is evidenced by acoustic streaming in the reactor base, several homogeneous grey regions, and regions of activity and inactivity which span over several wave lengths, indicating an already strong travelling wave. This suggests that an augmented travelling wave and diminished standing wave under flow only partly explain the photographic observations.

In other work, visualisation of the recirculating fluid showed that increasing flow rate pushed the incoming fluid further across the diameter of the transducer, before it mixed with the initial reactor fluid [28]. Thus, here, at no- or low-flow, mixing of the inlet and existing reactor fluids was to the left of the reactor and transducer centre. Then, as flow increased to 214.2 ml min^−1^, the mixing region moved towards the transducer centre, leading to disruption of quasi-acoustic streaming (lower red dashed box, Fig. 4). At higher flows, the mixing region was moved further right, past the transducer centre, and thus reduced disruption to streaming. There is further evidence of this in Fig. 4, as the brightness on the right-hand side of each photo increases with the onset of flow. The flow inlet and direction were perpendicular to the direction of sonication, and hence parallel with the pressure antinodes. Thus, flow pushed some SCL bubbles across the pressure antinodes to the right-hand side of the reactor. Once inside the reactor, the flow was upward, such that high flow rates pushed SCL bubbles to the outlet orifice or liquid surface, reducing the right-hand side brightness. This was likely not seen in the SL images (Fig. 3), since SL bubbles are larger than SCL bubbles [54], and therefore more stable under flow.

**Fig. 7:**
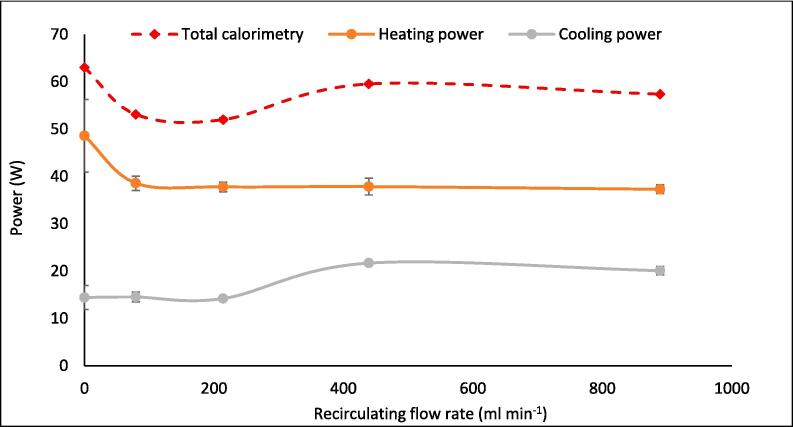
Breakdown of calorimetric measurements into heating and cooling power (W) vs recirculating flow rate (ml min^−1^). Data captured every 5 min during 30 min of sonication under 410 kHz and 80 W ultrasound. Error bars represent the standard deviation of three repeats.

Calorimetric power varied more significantly with flow (21.2 %) than fluoride release, dosimetry, SL, or SCL (and with reduced statistical overlap, Fig. 6). The relative calorimetric trend was the inverse of F^−^ release rates, with a 15.7 % decline upon flow addition. This, alongside the SL trend, suggests that thermal degradation is also not the primary cause of PFOS defluorination. Calorimetric power was divided into constituent heating and cooling rates (Fig. 7). The heating rate was reduced by 20.6 % with the onset of flow and remained near constant as flow increased further. Meanwhile, the cooling rate was near-constant, before increasing between 214.2 ml min^−1^ and 439.2 ml min^−1^. Thus, heat flux through the reactor walls, lid, transducer, and pump tubing was enhanced at some onset flow rate between these values, possibly by reduction of a static, insulating, boundary layer of fluid near the reactor/tubing walls (forced convection [46]). At 79–214 ml min^−1^, total calorimetric power reduced significantly (compared to no-flow) without an equivalent rise in cooling power until 439 ml min^−1^. The same power was applied to the transducer in all tests, hence, the “lost” calorimetric power must have been converted into some other form of power, such as fluid motion [55] or sonochemical activity. This qualitatively correlates with the increased F^−^ release rates, SCL intensity, and SCL activity/inactivity homogenisation at these flow rates.

### 3.3 Explanation of rate effects

A peak in sonochemical activity at intermediate flow rates agrees with other experimental works (KI dosimetry [24], [28], [29], SCL [32], [36]). Note that sonochemical activity encompasses many possible reactions involving; hydroxyl radicals (OH^•−^), superoxide radicals (O_2_^•−^), hydrogen superoxide radicals (HO_2_^•−^), H_2_O_2_, high temperatures, and solvated electrons (e^−^_aq_) [56]. These reactions respond differently to changes in reactor configuration and flow rate [30]. Given the inverse trends of SL, dosimetry, and calorimetry with F^−^ release rates, sonothermal and sono-radical mechanisms are unlikely dominant causes for PFOS degradation. Reduced I_3_^−^ formation rates at 79.2- and 214.2 ml min^−1^ (Fig. 5) indicate a loss of overall (OH^•−^ + O_2_^•−^) radical production rate [48]. Yet, SCL intensity is proportional to the rate of luminol excitation by O_2_^•−^ formation [57] and correlated well with F^−^ release rates (Fig. 2) and the inverse of I_3_^−^ production rates. O_2_^•−^ forms via reduction of molecular oxygen by an electron [58] or the removal of hydrogen from HO_2_^•−^ [48]. Flow likely enhanced air (and hence oxygen) sparging into the system [59], [60] and perhaps enhanced O_2_^•−^ production. However, SCL intensity did not increase continuously with flow (Fig. 2), as might be expected from the increased sparging. Hence, increased SCL intensities indicate enhanced e^−^_aq_ formation rates. Indirect measurements support e^−^_aq_ formation during single bubble SL [61], single bubble SCL [62], and multi-bubble SCL [63]. In multi-bubble systems, e^−^_aq_ do not appear in significant quantities, compared to radical species, such as H^•−^ and OH^•−^ [61]. Hence, a small increase in solvated electron release would enhance O_2_^•−^ production and thus SCL, while OH^•−^ production could reduce in greater measure, thus reducing I_3_^−^ production. Enhancement of e^−^_aq_ production would also be shared between reactions with molecular oxygen, PFOS, positively charged hydrogen, and metal ions [61] (K^+^ present here due to K-PFOS use), and likely several other species. Hence, the impact of enhanced e^−^_aq_ production on any one metric would be limited, as evidence by the small SCL intensity changes. Note that SL intensity cannot be subtracted from that of SCL for this work (to observe absolute SCL changes) since the two data were recorded under different photometric conditions (Section 2.4.3). Since fluoride release from PFOS was also maximised at 214.2 ml min^−1^ flow, this suggests that e^−^_aq_ could be the main driving force for PFOS sonolysis under the conditions used here, as suggested previously [9].

The causes of altered e^−^_aq_ and radical formation (and hence PFOS defluorination) rates may be related to the observed flow effects on bubble and wave dynamics (Section 3.2). As discussed, an enhanced traveling wave component under flow partly explains the homogenisation of light and dark SCL regions (Fig. 4) [28]. Enhanced travelling waves can also increase SCL intensity and reduce SL intensity, as observed at 214.2 ml min^−1^ (Fig. 2). Travelling waves augment asymmetric bubble collapse, leading to sonochemical effects (such a sonochemiluminescence), while standing waves are associated with symmetric collapse and thermal excitation leading to sonoluminescence [55]. Flow can reduce bubble sizes, due to reduced bubble coalescence [36]. Since SCL requires smaller bubbles than SL [54], this enhances SCL intensity and reduces SL intensity [36]. Smaller bubbles may have also enhanced the PFOS defluorination rate, as the population of cavitation nuclei increased [35], and total bubble surface area for PFOS adsorption increased. Further, asymmetric collapse can enhance non-volatile species injection into the bubble vapour and reduce collapse temperature [37], [64]. This would also reduce SL intensity and calorimetry with the onset of flow (Fig. 2 and Fig. 6). Further, high temperatures are needed to convert H_2_O to OH^•−^ [65]. Hence, the quenching of collapse temperatures by flow-induced asymmetry and reduced bubble size explains the reduced OH^•−^ and, hence, I_3_^−^ production rates. In a single bubble system, e^−^_aq_ production was only observed for a moving bubble, attributed to asymmetric cavity collapse, allowing microjet injection of non-volatile species into the bubble core [61]. e^−^_aq_ are relatively short lived (≤100 ps) [66] and would be most prominent in the collapsing bubble plasma core and interfacial region. Since PFOS is non-volatile [67] it relies on bubble surface population for degradation [1], [9], [10]. Therefore, flow likely enhanced e^−^_aq_ production as well as PFOS and O_2_ exposure to the bubble core, via increased bubble asymmetry. In turn, this enhanced the frequency of PFOS and O_2_ interaction with e^−^_aq_, leading to both increased PFOS defluorination and SCL.

At higher flow rates, bubble collapse temperatures were sufficiently reduced, by further asymmetry and non-volatile species injection, to counter the enhanced sonochemical activity seen at 79.2–214.2 ml min^−1^. This is evidenced by the reduced defluorination rate (Fig. 1), SCL intensity (Fig. 2), and calorimetric heating power (Fig. 7) at high flow rates. A sufficient reduction in collapse temperature would quench not only calorimetric power but also the plasma conditions in the bubble core, which would also affect radical and e^−^_aq_ production [61], [62], [63]. However, the SL intensity (Fig. 2) and I_3_^−^ production rate (Fig. 5) were enhanced at flow rates higher than 214.2 ml min^−1^, but diminished compared to the no-flow condition. This suggests that negative effects of flow on these metrics were slightly reduced at higher flow rates. These effects correlate with increased SCL heterogeneity (Fig. 2), suggested to be caused by mixing of inlet and reactor fluids off-centre from the transducer (Section 3.2). Off-centre fluid mixing would permit increased quasi-acoustic streaming but also an increase the standing wave component. In turn, this would increase bubble size and coalescence [36], collapse symmetry and temperature [37], [64] and hence, calorimetric powers, SL intensities [54], [55] and I_3_^−^ production rates [65]. Enhanced bulk mixing, with increased flow, will also have contributed to the enhanced calorimetric cooling power (due to forced convection [46]) and possibly augmented I_3_^−^ production by increasing the frequency of I^−^_(aq)_ interaction with radicals produced. While likely limited, bulk mixing effects are discussed further in Section 3.4.

Besides collapse intensity, flow and mixing can reduce bubble sizes, degassing, and reactor dead zones by reducing bubble coalescence via secondary Bjerknes forces [27], [34], [36]. Secondary Bjerknes forces decrease with increasing frequency [36], and mid-high ultrasonic frequencies (100–1000 kHz) show little response to increasing flow rates [30], [31], [32], as seen here. Further, high-power ultrasonic bubbles are sufficiently large that coalescence to larger sizes reduces SL and SCL activity, while lower-power bubbles are smaller, and coalescence increases their size sufficiently to generate sonochemical activity [36]. Since the reactor was already optimised for mid-high frequency [9] and moderate power (under review), sonochemical dead zones and excessive Secondary Bjerknes forces were limited, and mixing would have been of little benefit. Thus, the power and frequency used here were likely critical to the limited effect of flow on sonochemical phenomena [29]. However, initial bulk mixing also played a role, as discussed next.

### 3.4 Effects of flow over time

**Fig. 8:**
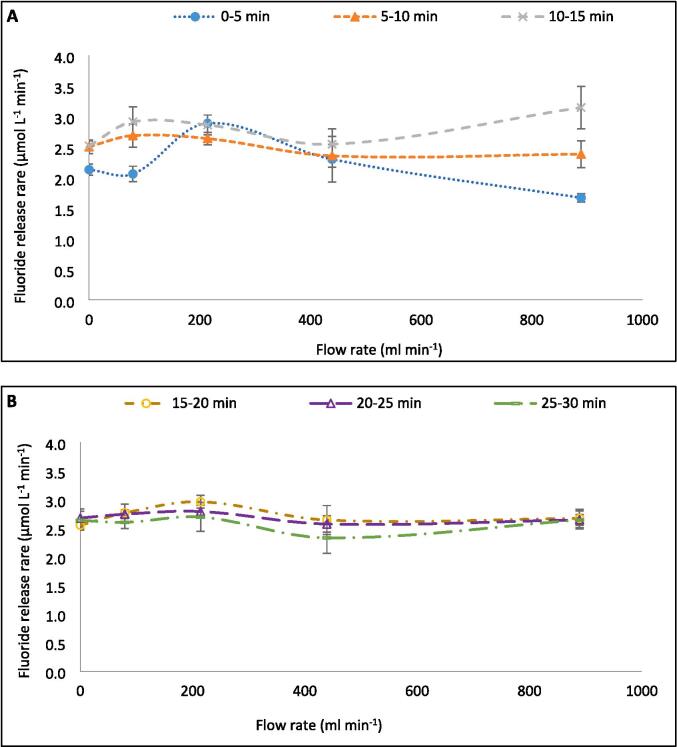

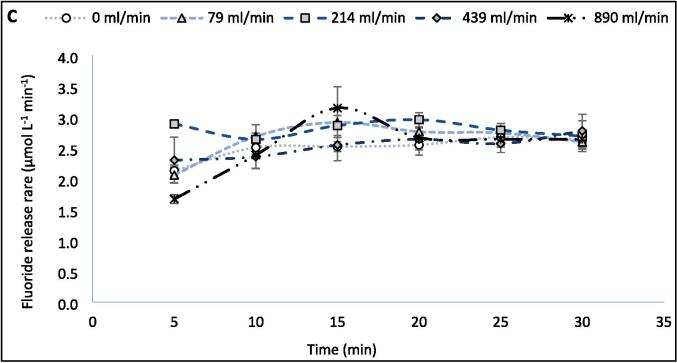
Variation of F- release rates (mol L^−1^ min^−1^) with increasing recirculating flow rate (ml min^−1^) over five-minute intervals during 30 min of PFOS sonolysis under 410 kHz and 80 W ultrasound per reactor. Data is separated into the first (A) and second (B) halves of the reaction time and variation by time with different flow rates (C). Error bars represent the standard deviation of three repeats.

For Section 3.1, the F^−^ production rates (and other sonochemical metrics) were measured every five-ten min during the 30-min reaction and averaged. However, when comparing the F^−^ release in five-min intervals (Fig. 8A–C), different trends are seen with increasing flow rate. Over 30 min (Fig. 1), the maximum fluoride release rate was 2.81 μmol L^−1^ min^−1^ (at 214 ml min^−1^) and the minimum was 2.46 μmol L^−1^ min^−1^ (at 79 ml min^−1^), giving a variation within 14.2 % of the minimum with varied flow rate. However, within the first 5 min of treatment, a maximum rate of 2.89 μmol L^−1^ min^−1^ was observed alongside a minimum of 1.67 μmol L^−1^ min^−1^, giving 73.1 % variation from the minimum. Variations in F^−^ release rate from the minimum in the 0–5, 5–10, 10–15, 15–20, 20–25, and 25–30-minute intervals are 73.1 %, 14.0 %, 24.8 %, 16.1 %, 8.5 %, and 15.6 %, respectively. In the first half of the reaction, the rate variation with flow is 37.3 %, while in the latter half it is 13.4 %. Fig. 8C shows the rate data against time, for each flow rate, and further demonstrates that the effect of different flow rates reduces as the reaction continues. This trend further explains the limited effect of flow seen during 30 min of sonication (Fig. 1). Hence, if a different treatment time was selected, the effects of flow might be stronger/weaker and an alternative optimum may have been found. Similarly, given the small effect of flow, alteration of other conditions, like PFOS concentration, would likely have changed the optimum flow condition.

A possible cause of the change in flow effects with time is the level of homogeneity in the reactor. In prior work, a 100 ml sonochemical reactor operated at 500 kHz behaved like a continuously stirred tank reactor (CSTR) regardless of the power used [25]. That is to say that sonochemical reactors are well mixed from the onset of the ultrasound, likely due to micro- and acoustic-streaming, which occur around small objects in the path of the sound wave, including bubbles [24], [68]. When the ratio of fluid volume to volumetric flow rate was reduced, the time to reach homogeneity was also reduced [25], likely due to increased randomness in flow direction and speed [27]. So, dynamic properties are impacted by flow addition. If homogeneity were a driving factor for the PFOS defluorination rate, a well-mixed reactor would have a high and near-constant reaction rate, as seen for the 214.2 ml min^−1^ data (Fig. 8C). Conversely, if homogeneity were the only relevant factor, then one would expect the highest flow rate to be the most well mixed [46] and show the highest and most consistent rate, which was not seen here. Thus, the effects of bulk mixing are complimented by effects described in 3.2, 3.3 (bubble size, asymmetric collapse, collapse temperature, nucleation rates, electron production, relative radical production rates, and exposure of PFOS to the bubble core).

**Fig. 9:**
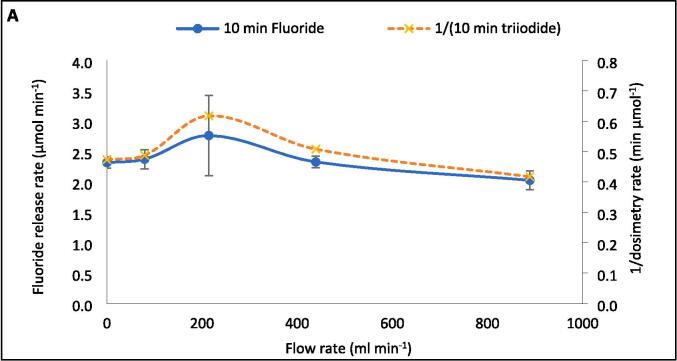

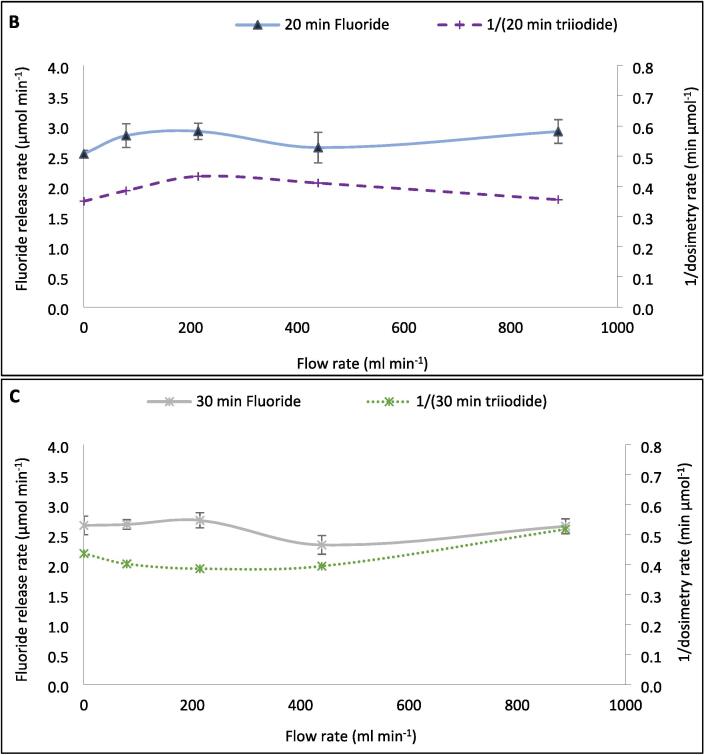
Variation of F- release rates (mol L^−1^ min^−1^) and the inverse of I3- generation rate (L min mol^−1^) with increasing recirculating flow rate (ml min^−1^) over 10-minute intervals during 30 min of PFOS sonolysis under 410 kHz and 80 W ultrasound. The data is separated into the 0–10 (A), 10–20 (B), and 20–30 (C) minutes of the reaction time. Error bars represent the standard deviation of three repeats.

The dosimetry data was taken every 10 min and the inverse of I_3_^−^ generation rate compared to the 10-min fluoride release rates (Fig. 9A–C). The relative changes in both metrics with increasing flow rate showed good agreement in the initial 10 min of sonication (Fig. 9A). However, as the reaction proceeded into the 10–20 min and 20–30 min periods (Fig. 9B-C, respectively), the relative agreement reduced. This demonstrates that the effect of flow on the rate of radical generation is dynamic with time, like the effect on defluorination rate but with an alternative trend. This may be partly due to the different times between samples (5 and 10 min, respectively) for the fluoride and dosimetric measurements. More frequent stoppage will have allowed for more bubbles trapped in the standing wave, to escape the reactor, and less opportunity for coalescence of bubbles to reach or exceed a sonochemically active size. This would have limited the bubble size range and thus the effect of flow on fluoride release. Variations in F^−^ release rates in the 0–10, 10–20-, and 20–30-min periods with increasing flow rate are 36.3 %, 14.8 %, and 17.7 %, respectively. Meanwhile, those of 1/[I_3_^−^ generation rate] are, at all times, larger (48.0 %, 23.5 %, and 34.0 %, respectively). However, as demonstrated in 3.2, 3.3, the mechanisms which contribute to fluoride release and dosimetry are clearly different and differently impacted by flow. Thus, the stoppage time is not the only factor at play. It is possible that the effect of flow on dosimetry is also diminishing with time, and that it may further reduce given an extended sonication time beyond 30 min.

**Fig. 10:**
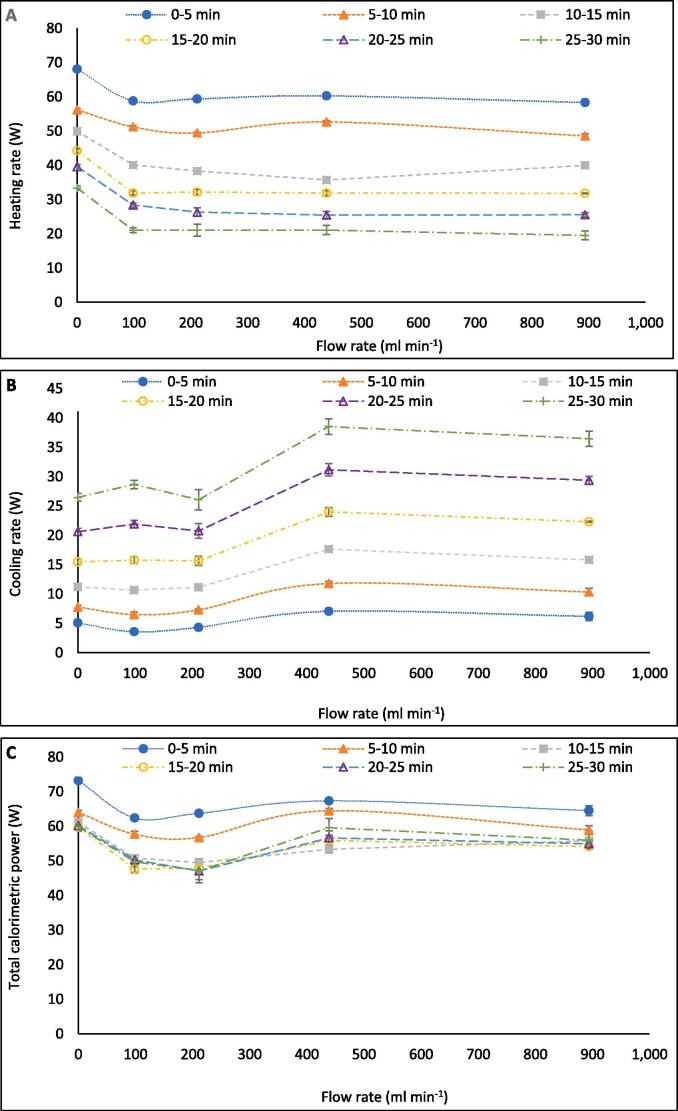
Variation of (A) heating, (B) cooling and total calorimetric (C) rates with increasing recirculating flow rate (ml min^−1^) over 5-minute intervals for 30 min sonication under 410 kHz and 80 W ultrasound. Error bars represent the standard deviation of three repeats.

Fig. 10A–C compare the changes in heating (A), cooling (B), and total calorimetric (C) power with flow rate over the 30-min sonication period, in 5-min intervals. As might be expected, the heating rate reduces with time as the reactor heats and the driving force for heat flux through the reactor walls is increased, hence increasing the cooling power. The relative shape of the heating profile stays near-constant over the 30-min period, while that of the cooling profile is exacerbated. This is likely due to the continued reduction of the static fluid layer at the reactor/pump-tubing walls with time [46]. However, as shown by Fig. 10C, the overall calorimetric profile changes within the first 0–15 min and then remains near static, suggesting that the factors controlling heating and cooling are in equilibrium after 10–15 min.

**Fig. 11:**
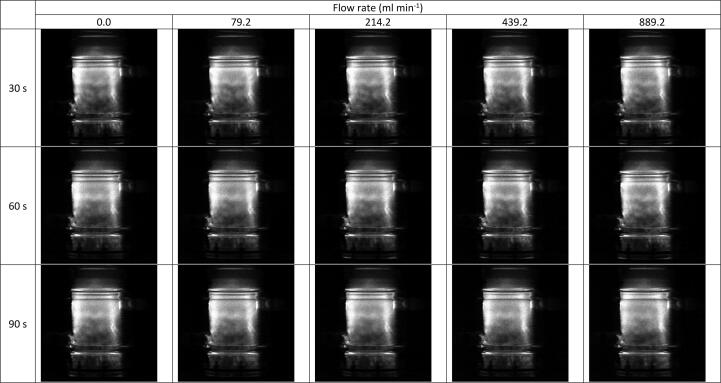
Sonoluminescence images for the sonolysis of 420 ml of Milli-Q water using 410 kHz ultrasound at 200 W L^−1^ applied power, at liquid flow rates of 0.0, 79.2, 214.2, 439.2, and 889.2 ml min^−1^, left to right respectively, and times of 30, 60, and 90 s, top to bottom respectively.

**Fig. 12:**
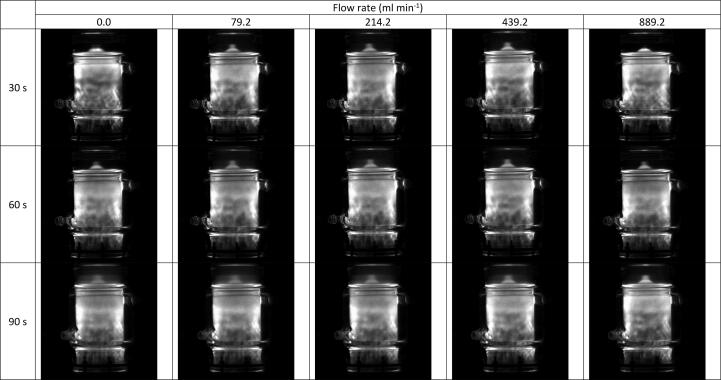
Sonochemiluminescence images for the sonolysis of 420 ml of Milli-Q water using 410 kHz ultrasound at 200 W L^−1^ applied power, at liquid flow rates of 0.0, 79.2, 214.2, 439.2, and 889.2 ml min^−1^, left to right respectively, and times of 30, 60, and 90 s, top to bottom respectively.

**Fig. 13:**
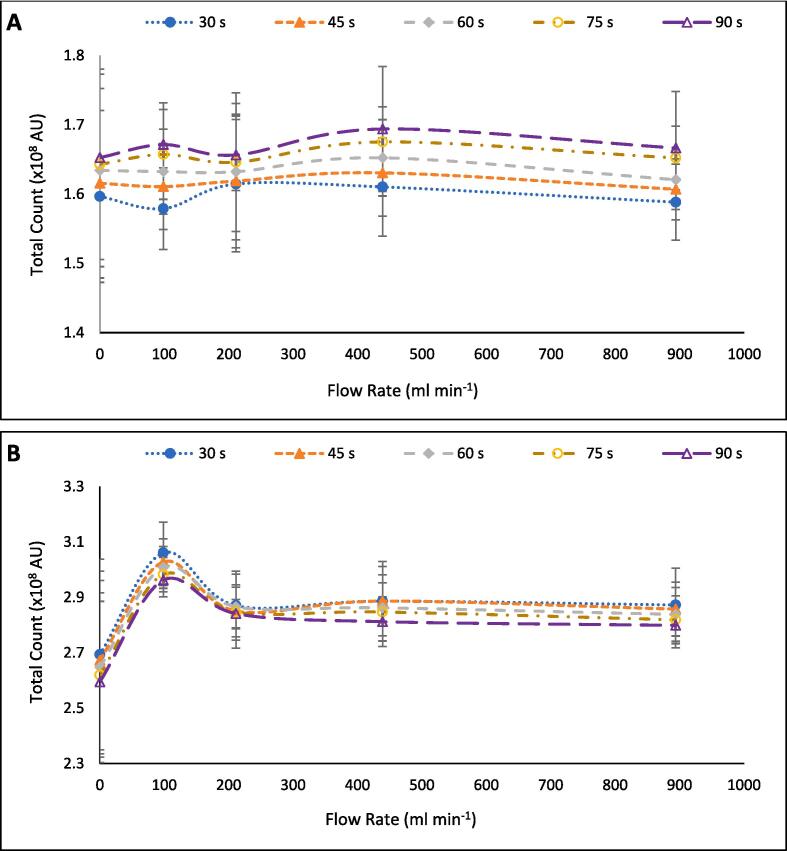
Sonoluminescence (A) and sonochemiluminescence (B) intensity for the sonolysis of 420 ml of Milli-Q water using 410 kHz ultrasound at 200 W L^−1^ applied power, at liquid flow rates of 0.0, 79.2, 214.2, 439.2, and 889.2 ml min^−1^ and times of 30, 45, 60, 74, and 90 s.

The SL/SCL data was taken after 30 s sonication time, since SL and SCL intensities can vary during the initial seconds of the reaction, but quickly stabilise [29], [30]. This was thought to be due to stabilisation of bubble populations, as larger bubbles would be degassed within the initial few seconds of sonication. However, the temporal measurements of fluoride (Fig. 8), dosimetry (Fig. 9), and calorimetry (Fig. 10) suggest bubble distributions continue to evolve over several minutes. It was not possible to fit the chiller inside the light-sealed box used for SL/SCL image capture. Hence, images over the 30-min sonication period could not be captured since the temperature would have increased up to 70 °C (Table S2) and thus affect results. However, images were captured after 30, 45, 60, 75, and 90 s, where the change in temperature would be minimal. Fig. 11 and Fig. 12 show these images for 30, 60, and 90 s and Fig. 13 shows the intensity data. While the SL images vary little, there is some reduction in the contrast of the image, as the contrast between the “anchor”-shaped black region and light regions at each flow rate appears to diminish with time (Fig. 11). A similar, yet more obvious, trend is seen with SCL images (Fig. 12) as the contrasting light and dark regions appear to blur into grey with time. The blurring is likely due to an increased proportion of travelling to standing wave in the reactor, which correlates well with theories of increased asymmetric collapse [37], [55] and enhanced sonochemical activity [29]. The difference in SL and SCL responses further suggests that the position of SL bubbles is less impacted by flow than SCL bubbles due to their larger size [54]. Further, the change in SCL contrast with time appears least significant for the images captured at 214 ml min^−1^. This suggests there is already a well homogenised bubble population which varies little with time, which agrees with the fluoride release rates (Fig. 8).

In Fig. 13, the equilibrium time for both SL and SCL bubble populations under flow appears longer than the assumed 30 s, as both data show minor changes over time. However, there is considerable statistical overlap between the data, with a maximum 5.5 % change in average SL intensity over the 90 s (79.2 ml min^−1^) and 3.2 % change for that of SCL (also 79.2 ml min^−1^). Prior work demonstrated that SL intensity was quasi-stable after just 0.3 s, oscillating around a peak value [69]. Hence, the oscillations after quasi-stabilisation could explain differences in images/intensity seen here, especially in the no-flow case. Note the prior study utilised 515 kHz pulsed ultrasound with on:off times of 4:12 ms and intensity of 0.20 W cm^−2^ without flow to sonicate 1.0 mM sodium dodecyl sulfate (a surfactant), compared to continuous 410 kHz ultrasound with an intensity of 2.27 W cm^−2^ and flow of 0–889 ml min^−1^ of pure water, used here. The former work also discussed that shorter pulse off times and lower surfactant concentrations hasten the time to reach peak SL intensity [69]. This suggests that the continuous ultrasound and lower surfactant (PFOS) concentration used here would have enabled peak sonoluminescence sooner than 0.3 s, at least in the no-flow case. These points suggests that photos or (intensity measurements) taken later in the reaction would likely not be statistically different to those in Fig. 11 and Fig. 12 and that the use of a 2 min sample time here would not have impacted the bubble populations for more than a few μs. Further work is needed to confirm whether SL and SCL measurements, here, were temporal after several minutes. Intriguingly, the SL intensity grows with time, while the SCL intensity decreases. Since the reactor increased in temperature by an average of 8.1 °C across all repeats and flow rates (Table S1), bubble sizes could have increased during the initial few minutes of sonication. Further, since SL bubbles are larger than SCL bubbles [54], the growth of SL bubbles likely enhanced intensity while SCL activity was quenched by the increase in size and loss of bubble surface area for electron ejection and interaction with O_2_.

The discussion on flow effects over time and the S(C)L images in Fig. 11 and Fig. 12 suggest that the main factors influenced by flow are dependent on a spatial–temporal bubble and reactant distribution, which is homogenised with time. The width of the size distribution of ultrasonic cavitation bubbles is inversely related to the frequency [54]. At 575 kHz, the distribution of cavity sizes leading to SCL was narrow and in the range of ≈ 2.6–4.2 μM [54]. Thus, under the 410 kHz ultrasound used here, there may be a slightly wider initial distribution of bubble sizes throughout the reactor. When the ultrasound is turned on, larger bubbles may be degassed, and smaller bubbles coalesce or dissolve into solution [70], contributing to a smaller bubble size range, hence, the limited impact on SCL. However, flow reduces secondary Bjerknes forces, coalescence, and degassing [27], [34], [36] while enhancing bubble nucleation and transition to pressure anti-nodes via primary Bjerknes forces [36], microstreaming, and bulk mixing [35]. These factors would likely enhance bubble size distribution at the lower end. Further, a spout was also observed at the liquid surface which would inject bulk air from the reactor head space into the bulk liquid and increase the bubble size range at the higher end. Thus, over several minutes, an equilibrium would be achieved between the factors which augment and diminish bubble populations and bubble sizes.

The limited impact of flow on several of the sonochemical metrics used here (F^−^ release, SCL intensity, dosimetry, calorimetry, etc.) would likely be reduced further at higher frequencies and increased at lower frequencies, as seen experimentally [30], [31], [32]. It was also shown here that different sonochemical factors take different times to equilibrate, with F^−^ release and calorimetry taking around 15 min, while dosimetry takes 20 min or more. Such times are in agreement with measured equilibrium time of an ultrasonic reactor to a step input in reactant concentration, and thus the time to reach homogeneity [25]. The time for SL/SCL stabilisation could be as low as 30 s, however, further work is needed to accurately determine these values. Overall, the temporal data has significant implications for sonochemists, since when using flow, measured rates and other data depend on the total experimental time. Hence, future studies should consider both pre- and post-equilibrium measurements for fair comparison of different sonochemical parameters (frequency, power, temperature, concentration, etc.).

## 4 Conclusions

The effects of recirculating flow on the rate of PFOS defluorination by sonolysis were considered. Moderate flows (79–214 ml min^−1^) enhanced defluorination rates up to 14 %, while higher flow rates (439–889 ml min^−1^) brought the rate back to a level comparable to that without flow. The defluorination rate was then compared to several sonochemical measurements and showed correlation with SCL, but inverse correlation with calorimetry, dosimetry, and, to some extent, SL. These findings were in general agreement with prior work and indicated that flow perturbs bubble surfaces and collapse shapes from “smooth and symmetrical” to “rough and asymmetrical”. This asymmetric collapse likely enhances solvated electron production and/or the brings PFOS molecules closer to sites of electron production, thus enhancing defluorination. However, sufficiently high flows (and collapse asymmetry) likely quenched collapse temperatures and sonochemical intermediate production (solvated electrons, radicals, etc.). The decline in radical production but enhanced SCL with the onset of flow was likely due to relative changes in HO_2_^•_^ and O_2_^•_^ radical populations. The subsequent rebound in radical production at high flows was possibly due to enhanced mixing and reduced buffering of acoustic streaming by the incoming fluid. All sonochemical effects were also time dependent with flow, indicating that bubble populations and size distributions were dynamic until a certain equilibrium time was reached. The time to equilibrate could have significant impacts for assessment of sonochemical activity, since the time of action is different for each. Hence, researchers should consider both pre- and post-equilibrium measurements when using flow in ultrasonic reactors. The effects of flow were limited due to the dynamic, well mixed nature of the sonochemical reactor, and the power and frequency used (previously optimised) which literature suggests produce limited Bjerknes forces. Since the addition of the pump scarcely affected power consumption (+1.9 %), treatment efficiency was improved with moderate flow rates. Thus, a flow through system may be economical for industrial scale treatment, as is commonly done in water treatment industries. Future work should consider waste stream specific flow optimisation and whether flow effects are similar for other PFAS and PFAS in complex matrices, which may affect acoustic streaming and hence collapse symmetry differently to pure PFOS. Higher flow rates and different flow orientations would likely also encourage different sonochemical effects.

## Declaration of Competing Interest

The authors declare that they have no known competing financial interests or personal relationships that could have appeared to influence the work reported in this paper.

## Data Availability

Data is provided in the supplementary files.
